# Artificial Butter

**Published:** 1874-06

**Authors:** 


*PACIFIC MEDICAL AND SURGICAL JOURNAL—March*, 1874:

Artificial Butter.—The manufacture of this article is fairly established in San Francisco. It is made by removing the stearin from tallow by chemical process, and agitating the residual olein and margarin with sour milk, the flavor of which is imparted to the product. It is white when made, but color is given it by annato or carrot. An unsuspecting person would eat it without discovering that it is anything else than the genuine article. It is more friable than ordinary butter, and though perfectly sweet, appears to be a trifle less sapid. There is a strong prejudice against it on account of its being made from tallow. But the tallow is perfectly pure and sweet, such as is used in cooking under the unoffending name of suet. The process of manufacture is cleanly, and the product is, to say the least, as free from objection on the score of cleanliness as natural butter, the chemical constituents being the same. The price in our market is fifty cents a roll (2 lbs.), whilst the best dairy butter commands seventy-five or more.

